# Glucocorticoid Receptor-Deficient Foxp3^+^ Regulatory T Cells Fail to Control Experimental Inflammatory Bowel Disease

**DOI:** 10.3389/fimmu.2019.00472

**Published:** 2019-03-18

**Authors:** Lourdes Rocamora-Reverte, Selma Tuzlak, Laura von Raffay, Marcel Tisch, Heidi Fiegl, Mathias Drach, Holger M. Reichardt, Andreas Villunger, Denise Tischner, G. Jan Wiegers

**Affiliations:** ^1^Division of Developmental Immunology, Biocenter, Medical University Innsbruck, Innsbruck, Austria; ^2^Department of Obstetrics and Gynecology, Innsbruck University Hospital, Innsbruck, Austria; ^3^Department of Dermatology, University Hospital Zurich, Zurich, Switzerland; ^4^Institute for Cellular and Molecular Immunology, University Medical Center Göttingen, Göttingen, Germany; ^5^CeMM Research Center for Molecular Medicine of the Austrian Academy of Sciences, Vienna, Austria; ^6^Ludwig Boltzmann Institute for Rare and Undiagnosed Diseases, Vienna, Austria

**Keywords:** glucocorticoid, glucocorticoid receptor, Foxp3, regulatory T cell, transfer colitis, suppression

## Abstract

Activation of the immune system increases systemic adrenal-derived glucocorticoid (GC) levels which downregulate the immune response as part of a negative feedback loop. While CD4^+^ T cells are essential target cells affected by GC, it is not known whether these hormones exert their major effects on CD4^+^ helper T cells, CD4^+^Foxp3^+^ regulatory T cells (Treg cells), or both. Here, we generated mice with a specific deletion of the glucocorticoid receptor (GR) in Foxp3^+^ Treg cells. Remarkably, while basal Treg cell characteristics and *in vitro* suppression capacity were unchanged, Treg cells lacking the GR did not prevent the induction of inflammatory bowel disease in an *in vivo* mouse model. Under inflammatory conditions, GR-deficient Treg cells acquired Th1-like characteristics and expressed IFN-gamma, but not IL-17, and failed to inhibit pro-inflammatory CD4^+^ T cell expansion *in situ*. These findings reveal that the GR is critical for Foxp3^+^ Treg cell function and suggest that endogenous GC prevent Treg cell plasticity toward a Th1-like Treg cell phenotype in experimental colitis. When equally active in humans, a rationale is provided to develop GC-mimicking therapeutic strategies which specifically target Foxp3^+^ Treg cells for the treatment of inflammatory bowel disease.

## Introduction

Regulatory T cells (Treg cells) expressing the transcription factor Foxp3 maintain immune homeostasis by limiting antigen-specific immune responses and sustaining tolerance to self-antigens (1). Most Treg cells are generated in the thymus (tTreg cells) as a separate lineage at the CD4^+^ single-positive stage of thymocyte development. Peripheral Treg cells (pTreg cells) are induced from peripheral CD4^+^Foxp3^−^ T cells in the presence of TGF-beta, however, the pool size and function of these pTreg cells is not fully characterized, mainly due to the lack of useful markers to discriminate tTreg from pTreg cells (2).

Treg cell function is not mediated by one single common pathway, as many different mechanisms have been described including downregulation of costimulatory molecules (CD80/CD86) on dendritic cells, secretion of inhibitory cytokines or metabolic disruption of target cells. Beyond that, Treg cells seem to have the capacity to adjust their suppressive mechanism(s) to a particular immune or inflammatory context, although the signals driving *in vivo* Treg cell adaptation are not well-understood (3).

The original view that tTreg cells are terminally differentiated and phenotypically stable has been recently questioned. Some Treg cells may lose Foxp3 expression in autoimmune disease (“ex-Foxp3” cells), others, while maintaining Foxp3 expression, acquire a certain degree of plasticity which is illustrated by secretion of pro-inflammatory cytokines and reduced suppressive function (4). The molecular mechanisms that drive Treg cell plasticity as well as the functional consequences for autoimmune diseases are largely unknown.

Glucocorticoids (GC) are best-known for their successful clinical usage as anti-inflammatory and immunosuppressive agents, despite their high potential for serious side effects. While the potency of (synthetic) GC as negative regulators of immune and inflammatory effector molecules at higher doses is well-documented, the effects of endogenous GC on the immune response and T cells in particular are much less clear. GC suppress T cell activation, both indirectly by inhibiting dendritic cell function and directly by inhibiting TCR signaling (5). T cell-specific deletion of the glucocorticoid receptor (GR) revealed T cells as critical targets for endogenous GC to both limit clinical disease in an animal model for multiple sclerosis (6) and prevent lethal immunopathology in an animal model for toxoplasma infection (7). As both studies utilized the *lck* promoter to drive expression of Cre recombinase for conditional deletion of the GR, CD8^+^ cytotoxic T cells, CD4^+^ T helper cells, and Foxp3^+^ Treg cells were GR-deficient. Treg cell development, steady-state homeostasis and function may be affected by GC, although reports are controversial. Administration of GC has been shown to increase both the proportion and number of murine CD4^+^CD25^+^Foxp3^+^ Treg cells in peripheral lymphoid organs (8). In line with this observation is the finding that Treg cells are relatively resistant to GC-induced apoptosis *in vitro* (9). In contrast, GC dose-dependently reduced both the proportion and total number of splenic Treg cells after repeated GC administration (10, 11). Likewise, therapeutic treatment of MOG-induced EAE with GC slightly reduced splenic Treg cell number and reduced Foxp3 expression levels (6). Human Treg cells accumulate relative to conventional T cells (Tcon) upon treatment of several autoimmune diseases with GC as reported for multiple sclerosis (12), systemic lupus erythematosus (13) and rheumatoid arthritis (14).

While effects of exogenous GC on Treg cells are obvious but controversial, it is not known whether endogenous GC regulate Treg cell homeostasis, both under steady state and inflammatory conditions. Lck-Cre GRfl/fl mice that lack the GR in all T cells, reportedly have reduced numbers of Treg cells in the thymus and periphery, but Treg cell function was not tested (15). Moreover, Treg cell homeostasis may be affected by GR-deficient conventional T cells that can give rise to pTreg cells.

We therefore generated mice with a specific deletion of the GR in Foxp3^+^ Treg cells by crossing GRfl/fl (16) with Foxp3-Cre mice (17). Remarkably, while Treg cell number, expression of Treg cell signature molecules, and *in vitro* suppression capacity of GR-deficient Treg cells was unchanged, GR-deficient Treg cells appeared defective in suppressing T cell-driven colitis in an *in vivo* mouse model for inflammatory bowel disease (IBD). This phenotype was associated with the acquisition of Th1 cell-like features in GR-deficient Treg cells. These data suggest that endogenous GC stabilize Treg cell fate and function under inflammatory conditions and provide a rationale for the development of GC therapy for IBD that specifically targets Treg cells and expectedly reduces the strong side-effects of these hormones.

## Results

### Verification of Specific GR Deletion in Foxp3^+^ Treg Cells

**Figure 1:**
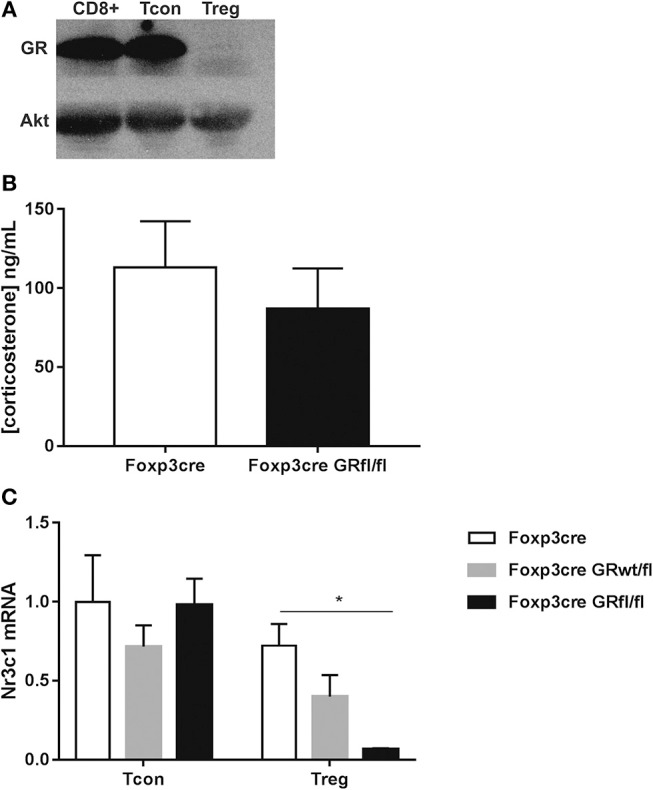
Physical characterization of GR deletion in Foxp3^+^ Treg cells. **(A)** Immunoblotting shows GR protein expression in purified CD8^+^, CD4^+^CD25^−^ Tcon, and CD4^+^Foxp3^+^ Treg cells from Foxp3-Cre GRfl/fl splenocytes. **(B)** Serum samples from Foxp3-Cre and Foxp3-Cre GRfl/fl mice were analyzed for corticosterone content by ELISA. **(C)** Real time qPCR analysis of *Nr3c1* (GR) mRNA expression in CD4^+^CD25^−^ Tcon and CD4^+^Foxp3^+^ Treg cells from Foxp3-Cre, Foxp3-Cre GRwt/fl and Foxp3-Cre GRfl/fl mice. Nr3c1 mRNA expression levels are referred to mRNA levels of CD4^+^ Tcon cells from Foxp3-Cre mice according to the ΔΔCt relative quantification method. Data are shown as mean ± SEM (*n* ≥ 3).

Mice carrying a specific deletion for the GR in Foxp3^+^ Treg cells (Foxp3-YFP-iCre x GRfl/fl mice; dubbed here: Foxp3-Cre GRfl/fl mice) developed normal and did not show any signs of disease. Lack of GR in Foxp3^+^ Treg cells was confirmed at the protein level both in spleen (Figure 1A) and thymus (Figure S1A). Ectopic recombination by Cre-YFP expressed under the control of the FoxP3 promoter of some conditional alleles (*Cd28*), but not others (*R26-RFP*), has been reported (18). However, quantification of the GR in conventional CD4^+^CD25^−^ Foxp3^−^ T cells, CD8^+^ T cells and B cells revealed no differences between wild type (WT), Foxp3-Cre and Foxp3-Cre GRfl/fl mice (Figure S1B), ruling out promiscuous Foxp3-Cre expression in these lymphocyte subsets. Since endogenous GC have been shown to regulate T cell numbers (19, 20), we determined peripheral blood levels of corticosterone in our mouse strains to check for potential differences. However, this appeared not to be the case as no differences in corticosterone levels were found (Figure 1B). Expression levels of *Nr3c1* (encoding the GR) by CD4^+^CD25^−^ Tcon cells and CD4^+^Foxp3^+^ Treg cells were quantified by qPCR. Splenic Treg cells from heterozygous Foxp3-Cre GRwt/fl mice expressed *Nr3c1* at approximately half of control Treg cells from Foxp3-Cre mice (Figure 1C). Finally, Treg cells derived from Foxp3-Cre GRfl/fl mice were resistant to *in vitro* corticosterone-induced cell death, confirming the absence of the GR at the functional level (Figure S1C). Thus, Foxp3-Cre GRfl/fl mice lack the GR specifically in Foxp3^+^ Treg cells with no signs of significant recombination in CD4^+^ Tcon cells or other lymphocyte subsets.

### Basic Immune Characteristics of Mice Lacking the GR in Treg Cells

**Figure 2:**
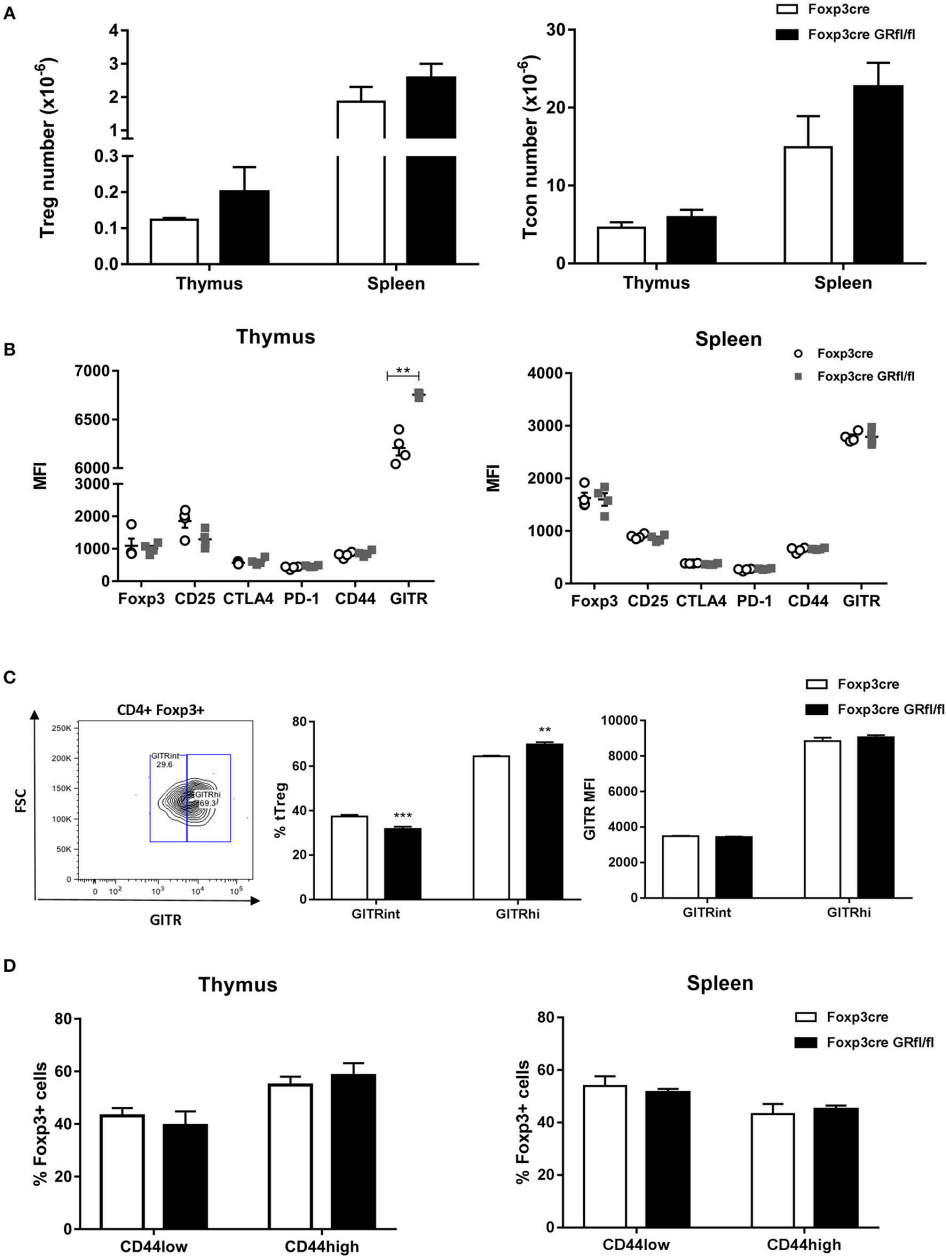
Immune characteristics of Foxp3-Cre GRfl/fl mice. **(A)** Thymi and spleens from Foxp3-Cre and Foxp3-Cre GRfl/fl mice were analyzed for cellularity of CD4^+^Foxp3^+^ Treg (left panel) and CD4^+^CD25^−^ Tcon cells (right panel). **(B)** Treg cell signature marker expression by thymic (left panel) and splenic (right panel) CD4^+^Foxp3^+^ Treg cells. Data shown are median immunofluorescence intensity values from individual Foxp3-Cre or Foxp3-Cre GRfl/fl mice. **(C)** Thymic Treg cells were divided into subsets according to their GITR expression levels (GITR^int^ or GITR^high^; left panel: gating; middle panel: frequency; right panel: MFI). **(D)** CD44 expression level of thymic (left panel) or splenic (right panel) CD4^+^Foxp3^+^ Treg cells from Foxp3-Cre and Foxp3-Cre GRfl/fl mice. Data are shown as mean ± SEM (*n* = 4).

Foxp3-Cre GRfl/fl mice showed normal CD4^+^Foxp3^+^ Treg (Figure 2A, left panel) and CD4^+^ Tcon (Figure 2A, right panel) cell numbers in the thymus and spleen. Next, we examined steady state expression of Treg cell signature molecules such as Foxp3, CD25, GITR, and CTLA-4 by Treg cells of Foxp3-Cre and Foxp3-Cre GRfl/fl mice. In both thymus and spleen, expression levels of these markers were comparable between GR-expressing and GR-deficient Treg cells, except for thymic GITR that showed significantly higher expression in Treg cells from Foxp3-Cre GRfl/fl mice (Figure 2B, left panel). Since GITR expression levels are critical for Treg cell maturation (21), we further analyzed CD4^+^Foxp3^+^ thymocytes for GITR^int^ and GITR^high^ expressing subsets. A moderately enhanced frequency of Foxp3^+^GITR^high^ and a reduction of Foxp3^+^GITR^low^ cells in Foxp3-Cre GRfl/fl mice was observed, as compared to control Foxp3-Cre mice (Figure 2C). The functional relevance of this observation is presently unclear, yet this suggests that basal GITR expression in splenic Treg cells is not dependent on a functional GR. Treg cells consist of both naïve CD44^low^CD62L^high^ and CD44^high^CD62L^low^ “effector-like” subpopulations, the latter exerting suppressor activity (22). We therefore analyzed the fractions of both Treg cell subsets in the context of GR-deficiency and found equal amounts in both Foxp3-Cre and Foxp3-Cre GRfl/fl mice (Figure 2D). The transcription factor Helios has been proposed as a marker to discriminate tTreg from pTreg cells (3). Examination of both fractions and Helios expression levels, however, revealed no changes between Treg cells from Foxp3-Cre and Foxp3-Cre GRfl/fl mice (Figure S2A). Finally, activation of CD4^+^ Tcon and CD8^+^ T cells by anti-CD3/anti-CD28 antibodies was comparable in Foxp3-Cre and Foxp3-Cre GRfl/fl mice regarding induction of CD44 and production of IFN-gamma (Figure S2B). In summary, deletion of the GR in Treg cells does not modify their basal cell number, phenotype or activation competence.

### *In vivo* Survival of Treg Cells Does Not Depend on GR Expression

**Figure 3:**
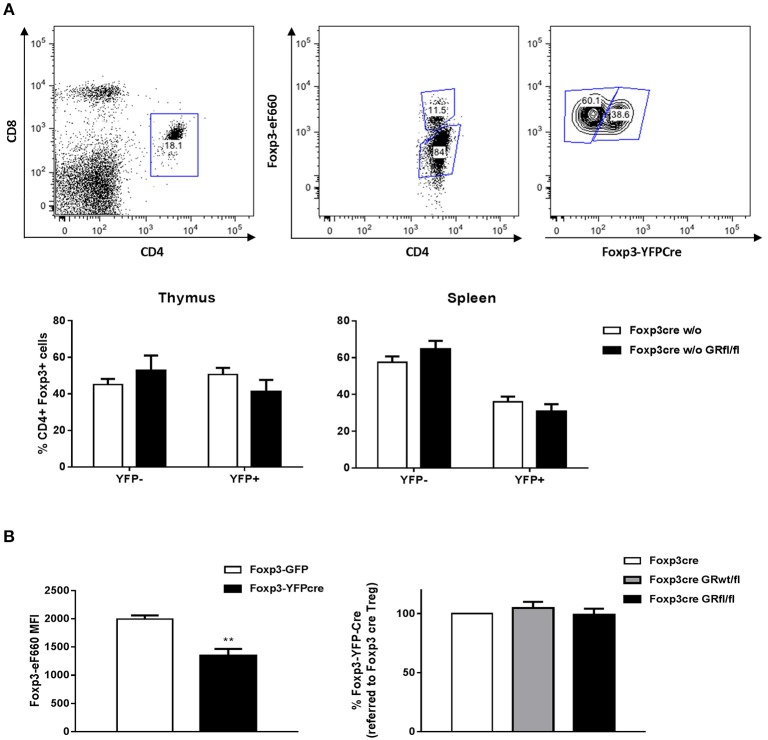
Treg cell survival does not depend on GR expression. **(A)** CD4^+^Foxp3^+^ Treg cells from heterozygous female Foxp3-Cre/wt and Foxp3-Cre/wt GRfl/fl mice were divided into YFP^+^ (Cre^+^) and YFP^−^ (Cre^−^) cells according to the gating strategy shown in the upper panels. Lower panels show YFP^+^ vs. YFP^−^ fractions in the thymus (left panel) or spleen (right panel). CD4^+^Foxp3^+^YFP^+^ Treg cells from heterozygous female Foxp3-Cre/wt GRfl/fl mice are GR-deficient whereas CD4^+^Foxp3^+^YFP^−^ Treg cells are GR-sufficient. **(B)** Foxp3 expression levels of Treg cells from WT (Foxp3-GFP reporter) and Foxp3-YFP-Cre mice (left panel). Data are shown as mean ± SEM (*n* = 3). The right panel shows Foxp3 expression in Treg cells from Foxp3-Cre, Foxp3-Cre GRwt/fl and Foxp3-Cre GRfl/fl mice. Data are shown as mean ± SEM (*n* = 5).

To directly assess the impact of GR deletion in Treg cells on their survival in a competitive setting, we generated heterozygous female Foxp3-Cre/wt GRfl/fl mice. As *Foxp3* is located on the X chromosome, random inactivation of one allele in these mice is predicted to produce 50% of Treg cells that use the WT allele (i.e., GR-sufficient) and 50% of Treg cells that use the Foxp3-Cre allele (i.e., GR-deficient). In spleen, but not in thymus, we observed a moderate competitive disadvantage of Treg cells expressing the Foxp3-Cre allele (Figure 3A), a finding previously reported by others (23). However, in both thymus and spleen, equal proportions of WT and GR-deficient Treg cells were generated and/or survived, suggesting that the GR does not influence survival of Treg cells in a physiologically normal setting (Figure 3A). The observation that the Foxp3-Cre allele may affect peripheral Treg cell survival, together with the finding that the Foxp3-Cre allele is mildly hypomorph as reported by others (18), prompted us to determine Foxp3 expression levels in WT and Foxp3-Cre-expressing mouse strains. In agreement with Franckaert et al. (18) we found a ~30% reduction of Foxp3 protein expression in mice expressing the Foxp3-Cre allele as compared to WT mice (Figure 3B, left panel), whereas all mouse strains expressing the Foxp3-Cre allele displayed equal amounts of Foxp3 (Figure 3B, right panel). Since we did not find deviations produced by the Foxp3-Cre allele other than those shown in Figure 3, mice expressing this allele were used as controls in our experiments (Foxp3-Cre mice). Finally, to test for potential epigenetic changes in critical regions of the *Foxp3* locus we analyzed the methylation status as described previously (24). However, the degree of methylation of CpG islands within the *Foxp3* locus appeared comparable in WT, Foxp3-Cre, and Foxp3-Cre GRfl/fl mice (Figure S3), supporting the finding that Foxp3 expression is unchanged in the absence of the GR (Figure 3B). Hence, *in vivo* survival of Treg cells and expression of their lineage specification factor Foxp3 is, at least under basal conditions, not dependent on expression of the GR by these cells.

### Antinuclear Antibody Prevalence Is Increased in Foxp3-Cre GRfl/fl Mice

**Figure 4:**
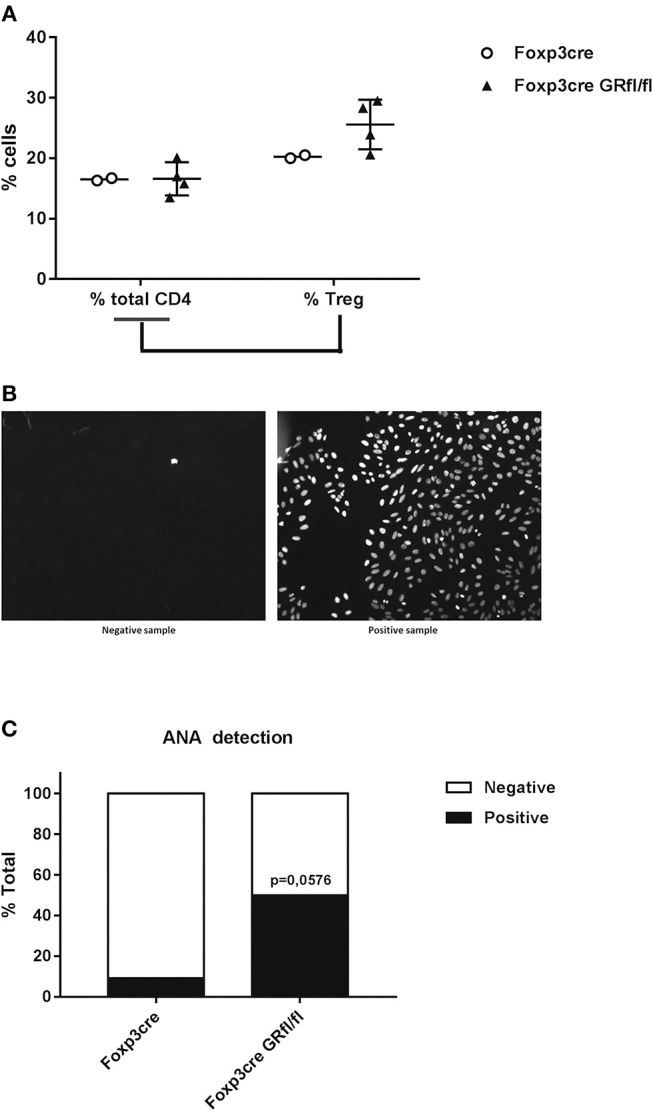
Increased frequency of antinuclear antibodies (ANA) in Foxp3-Cre GRfl/fl mice. **(A)** Flow cytometry analysis of splenic total CD4^+^ and CD4^+^Foxp3^+^ Treg cells (expressed as a percentage of total CD4^+^ cells) from Foxp3-Cre and Foxp3-Cre GRfl/fl mice. **(B)** Example of ANA determination by immunofluorescence. Mouse sera were incubated with HEp-2 cells and the presence of ANA was determined by indirect immunofluorescence microscopy. The sample on the left is ANA negative, while the sample on the right is considered ANA positive. **(C)** Presence of ANA in sera from 8 to 13 months old Foxp3-Cre and Foxp3-Cre GRfl/fl mice. Data are shown as mean ± SEM (*n* ≥ 7).

Since both Treg cell number and function change with age, we analyzed 13 months old Foxp3-Cre and Foxp3-Cre GRfl/fl mice for splenic Treg cell number and found no major changes between these genotypes (Figure 4A; Figure S4). Scurfy mice, who are deficient for regulatory T cells, develop antinuclear antibodies (ANA) and lupus-like disease (25, 26). When sera of GR-deficient Treg cell mice were investigated for the presence of ANA, it appeared that higher fractions were positive as compared to Foxp3-Cre mice (Figures 4B,C). This observation appeared to be sex independent. Accordingly, Treg cell-intrinsic expression of the GR seems to prevent loss of tolerance to these autoantigens with age.

### Defective Function of GR-Deficient Treg Cells *in vivo* but not *in vitro*

**Figure 5:**
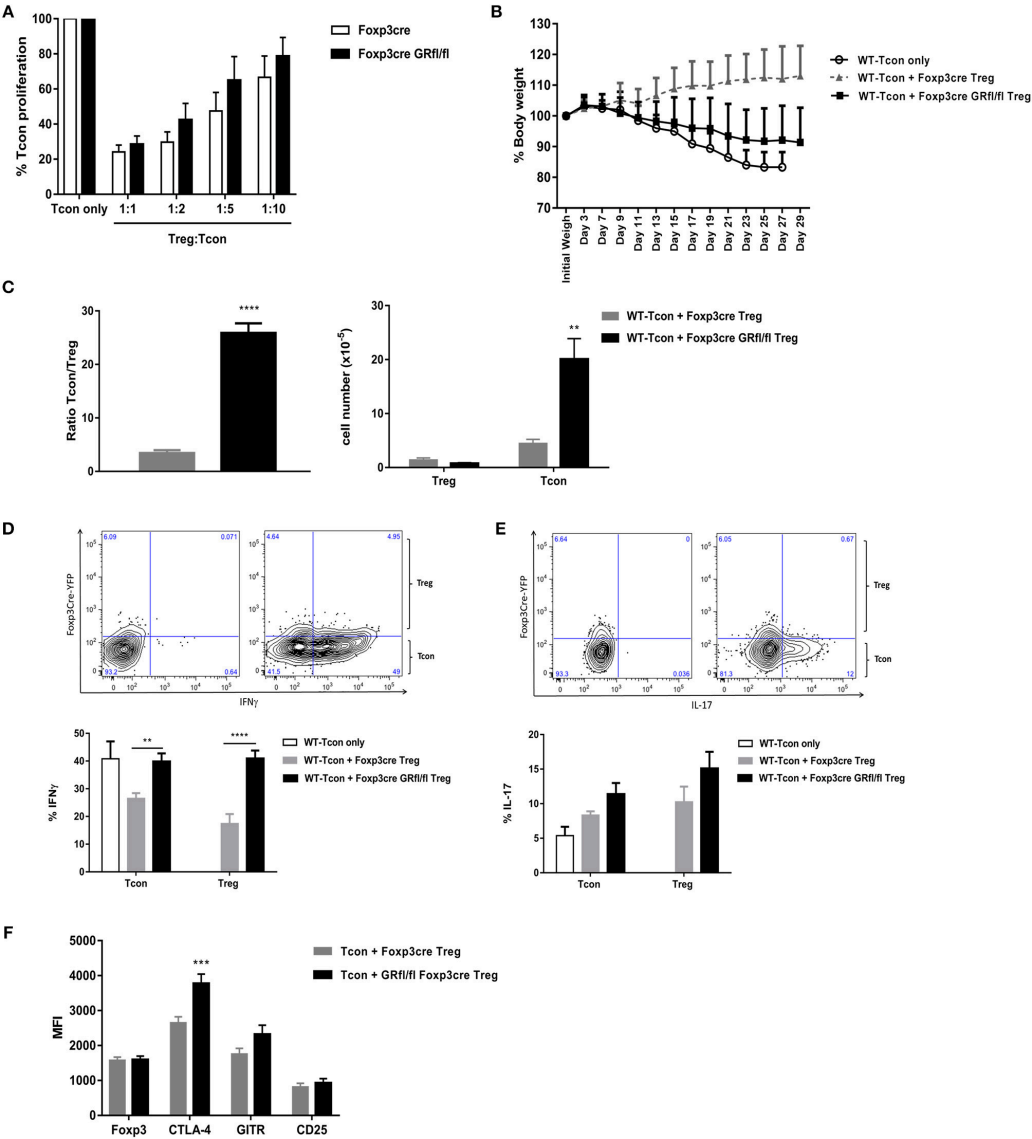
Suppression capacity of GR-deficient Treg cells is defective *in vivo* but not *in vitro*. **(A)** *in vitro* T cell suppression assay: WT CD4^+^Foxp3^−^CD25^−^CD45RB^high^ Tcon cells were cultured either alone or co-cultured at different ratios with CD4^+^Foxp3^+^CD25^+^CD45RB^low^ Treg cells derived from Foxp3-Cre or Foxp3-Cre GRfl/fl mice. Data are shown as mean ± SEM (*n* = 5). **(B)** T cell transfer model of colitis in RAG1^−/−^ mice. WT CD4^+^Foxp3^−^CD25^−^CD45RB^high^ Tcon cells were either transferred alone (WT-Tcon only) or co-transferred with CD4^+^Foxp3^+^CD25^+^CD45RB^low^ Treg cells from Foxp3-Cre or Foxp3-Cre GRfl/fl mice. Body weight was assessed over time and animals were sacrificed either when weight loss exceeded 15% or 4 weeks after cell transfer (day 29) **(C)** Splenic CD4^+^CD25^−^ Tcon and CD4^+^Foxp3^+^ Treg cells were enumerated (right panel) and the ratio between these subsets calculated (left panel). *In vitro* cytokine production of IFN-gamma **(D)** and IL-17 **(E)** by either splenic CD4^+^CD25^−^ Tcon or CD4^+^Foxp3^+^ Treg cells obtained from RAG1^−/−^ mice treated and sacrificed as described in **(B)**. Representative gating strategy for IFN-gamma [**(D)**, upper panels] and IL-17 [**(E)**, upper panels] shows unstained samples (upper left panels) and cytokine-stained samples (upper right panels), derived from a mouse receiving WT-Tcon + GR-deficient Treg cells. **(F)** Treg cell signature marker expression by splenic Treg cells taken from mice described in **(B)**. Data are shown as mean ± SEM (*n* ≥ 7).

The increased presence of ANA in our GR-deficient Treg cell mice prompted us to study the suppressive capacity of their Treg cells, first tested in an *in vitro* assay. Naïve CD4^+^ Tcon cells from Foxp3-Cre control mice were stimulated with anti-CD3 mAb in the presence of irradiated antigen presenting cells (APCs) and co-cultured with different Treg cell numbers derived from Foxp3-Cre or Foxp3-Cre GRfl/fl mice. Proliferation of Tcon cells, assessed after 3 days, was potently suppressed by Treg cells, however, Foxp3-Cre and Foxp3-Cre GRfl/fl Treg cells exhibited an equal inhibitory capacity (Figure 5A). Similar results were obtained when Treg cells from Foxp3-Cre or Foxp3-Cre GRfl/fl mice were compared for their ability to inhibit proliferation of CD4^+^CD44^+^CD62L^−^ memory T cells (Figure S5A).

Since many autoimmune-prone mouse strains carrying Treg cell specific mutations have normal Treg cell suppressor function *in vitro* (3), we set out for *in vivo* functional testing of GR-deficient Treg cells in a mouse model for inflammatory bowel disease, i.e., T cell transfer colitis in RAG1^−/−^ mice (27). These mice produce no mature T cells or B cells and develop colitis upon transfer of Treg cell-depleted CD4^+^Foxp3^−^CD25^−^CD45RB^high^ Tcon cells (WT-Tcon only; Figure 5B). Co-transfer of CD4^+^Foxp3^+^CD25^+^CD45RB^low^ Treg cells from Foxp3-Cre mice (WT-Tcon + Foxp3-Cre Treg) prevented, as expected, the development of disease. Strikingly, Treg cells derived from Foxp3-Cre GRfl/fl mice were largely ineffective under these experimental conditions (WT-Tcon + Foxp3-Cre GRfl/fl Treg; Figure 5B).

The failure of GR-deficient Treg cells to protect against colitis was also evident from histological assessment of intestinal inflammation (Figure S5B). Blinded grading of colonic inflammation revealed complete protection in mice treated with control Treg cells whereas suppression of intestinal inflammation in mice treated with GR-deficient Treg cells was incomplete.

Analysis of the Tcon:Treg cell ratio in spleens of RAG1^−/−^ mice sacrificed after 4 weeks revealed a striking 8-fold higher ratio in mice receiving Treg cells from Foxp3-Cre GRfl/fl, as compared to Foxp3-Cre mice (Figure 5C, left panel), indicating strong relative expansion of Tcon cells in the presence of GR-deficient Treg cells (Figure 5C, right panel).

Since expansion of Tcon cells is consistent with a pro-inflammatory phenotype, we next studied pro-inflammatory cytokine expression by splenic Tcon and Treg cells. The fraction of Tcon cells producing IFN-gamma after 4 weeks was highest in mice with the strongest disease symptoms, i.e., those receiving either Tcon cells (WT-Tcon only) or those co-injected with Tcon cells plus Treg cells from GR-deficient Treg cell mice (WT-Tcon + Foxp3-Cre GRfl/fl Treg; Figure 5D). Remarkably, significantly more Treg cells producing IFN-gamma (Figure 5D), but not IL-17 (Figure 5E), were present in mice that were treated with GR-deficient Treg cells than in mice receiving control Treg cells. Treg cell signature marker expression levels were similar between control and GR-deficient Treg cells (Foxp3, GITR and CD25), with the exception of CTLA-4 which was significantly elevated on GR-deficient Treg cells (Figure 5F and Figure S5C). Analysis of IFN-gamma producing Treg cells for Foxp3 expression levels revealed no significant differences between GR-deficient and GR-proficient Treg cells (Figure S5D).

Further in depth analysis of Treg cell markers and subsets was performed on splenic Treg cells that were used in the transfer colitis experiments, i.e., CD4^+^Foxp3^+^CD45RB^low^ cells (for gating, see Figure S6A) derived from Foxp3-Cre and Foxp3-Cre GRfl/fl mice, revealing no differences regarding expression levels of Foxp3, CD25, Latency Associated Peptide (LAP), Lymphocyte-activation gene 3 (LAG-3), PD-1 and GITR (Figure S6A, right panel). Fractions of CD4^+^Foxp3^+^CD45RB^low^ cells expressing these markers were also similar in both mouse strains with the exception of a reduction in PD-1 expressing cells (Figure S6A, middle panel). We next analyzed the presence of two recently described Treg cell subsets, i.e., GITR^high^PD-1^high^CD25^high^ (Triple^high^) Treg cells, which reportedly control *in vivo* lymphocyte proliferation, and GITR^low^PD-1^low^CD25^low^ (Triple^low^) Treg cells, which have been shown to limit colitis (28). Interestingly, while the fraction of Triple^high^ Treg cells appeared reduced in Foxp3-Cre GRfl/fl mice, Triple^low^ Treg cells were not significantly changed as compared to Foxp3-Cre mice (Figure S6B, lower left panel). In addition, mean expression levels of GITR, PD-1, and CD25 were similar between both mouse strains (Figure S6B, lower right panel).

A different Treg cell subset which may suppress colitis has the phenotype Foxp3^low^CD25^−^GITR^+^, designated GITR single-positive cells (29). A comparison of this subset in spleens from Foxp3-Cre and Foxp3-Cre GRfl/fl mice revealed, however, no significant differences (Figure S6C).

Taken together, our findings during experimental intestinal inflammation indicate that GR-deficient Treg cells, while retaining expression of Treg cell markers, acquired an increased plasticity toward a Th1-like Treg cell phenotype that was accompanied by a reduction in the suppressive capacity of these cells.

## Discussion

Here, we show that GR-deficient Treg cells, while displaying no changes in function under basal conditions, are defective under inflammatory conditions and gain the ability to produce effector cytokines that are characteristic for Th1-like Treg cells. In line with their general inhibitory properties during inflammation, endogenous GC are apparently required to prevent Treg cell plasticity that is associated with reduced suppressive function.

The finding that GR-deficient Treg cell mice have normal numbers of Treg cells in both thymus and spleen differs from a report by Mittelstadt et al. (15) showing reduced Treg cell numbers in both organs in mice deficient for the GR in all T cells (Lck-Cre GRfl/fl mice). However, the lack of the GR in non-Treg T cells in Lck-Cre GRfl/fl mice may account for this difference. Supporting the view that GC, at least under basal conditions, do not influence Treg cell homeostasis are the results of the competitive experiments in heterozygous female Foxp3-Cre/wt GRfl/fl mice, clearly showing that in a physiologically normal environment no differences were observed in development and/or survival between WT and GR-deficient Treg cells.

GC may regulate T cell number in both thymus and peripheral lymphoid organs but data from previous studies are conflicting and Foxp3^+^Treg cells were not specifically analyzed. Thymocyte number and subset distribution in different mouse strains targeting exon 2 has been shown to be unchanged (30, 31). Studies conditionally targeting exon 3 of the GR revealed either no changes in thymocyte numbers (32) or a clear reduction (15) without a change in major subset composition. Similar results were found for peripheral T cell numbers in both mouse strains. It is currently unclear why both mouse models targeting exon 3 display substantial differences with respect to the size of their T cell pools. Conversely, transgenic mice overexpressing the GR 2-fold selectively in T cells showed a reduction in both thymocyte and peripheral T cell numbers (19). Moreover, transfer of bone marrow cells from mice expressing a gain of function GR knock-in into irradiated WT mice revealed a strong reduction of T-cell numbers analyzed ten weeks later, as compared to irradiated mice that received bone marrow from WT mice (33). Collectively, these findings suggest that endogenous GC regulate T cell homeostasis to some extent and such control may be more pronounced once peripheral GC concentrations are elevated which reportedly occurs upon activation of the immune system (34).

Expression of Treg cell signature molecules by Treg cells were similar between Foxp3-cre and Foxp3-Cre GRfl/fl mice, with the exception of a small, but significant increase in frequency of GR-deficient thymic Foxp3^+^ cells expressing the TNF receptor superfamily member GITR at high levels. Since on the one hand GITR regulates Treg cell development and correlates with TCR signal strength (21, 35) and, on the other hand, GC reportedly induce GITR in T cell hybridoma cells (36), this observation seems counterintuitive at first sight. However, GC were shown to induce very limited upregulation of GITR in primary CD4^+^ T cells, whereas TCR signaling appears to be a much stronger inducer of GITR than GC (37). Moreover, TCR signaling in the presence of GC seems to reduce GITR expression in these cells as compared to TCR signaling alone (37). Our observation that GITR expression is increased in GR-deficient Treg cells suggests that endogenous GC may inhibit TCR-induced GITR in Foxp3^+^ Treg cells as well. According to the “mutual antagonism” hypothesis, crosstalk between GR signaling and TCR signaling leads to survival of conventional T cells bearing TCRs that build up a repertoire that is required for a robust adaptive immune response (15, 38). Whether GC also change the TCR repertoire of Treg cells and, by this means, affect their functional competence, remains to be established. Our finding that splenic GITR^high^PD-1^high^CD25^high^Foxp3^+^CD45RB^low^ Treg cells were reduced in Foxp3cre GR/fl/fl mice does not point to an increased TCR affinity for self-antigens of these GR-deficient Treg cells, at least in the periphery.

While Treg cell signature molecule expression and the suppressive capacity of GR-deficient Treg cells on *in vitro* Tcon cell proliferation appeared unaffected, the increased presence of ANA in Foxp3-Cre GRfl/fl mice at older age provided the first indication for a potentiating role of the GR in Treg cell function. Since we did not observe significant changes in both the percentage and the absolute Treg cell number in our mouse strains, we assume that the functional competence of Treg cells decreases with age in the absence of cell-intrinsic GR expression. Alternatively, in the GR-deficient Treg cell population, we detected a reduced fraction of GITR^high^PD-1^high^CD25^high^ Treg cells, which reportedly inhibit *in vivo* lymphocyte proliferation (28). However, whether this observation contributes to the increased presence of ANA in Foxp3-Cre GRfl/fl mice at older age is currently not known. The second observation that the GR is required for full Treg cell function was made in the transfer colitis model. Our findings suggest that under inflammatory conditions Treg cells that lack the GR may become unstable regarding their suppressive regulatory T cell function. Moreover, it seems that GR-deficient Treg cells gained the ability to produce effector cytokines that are characteristic for Th1 cells. Such Th1-like Treg cells producing IFN-gamma (but maintaining Foxp3 expression) have been reported to be present at an increased frequency in both mouse models (39) and patients with autoimmune diseases such as type 1 diabetes (40) or multiple sclerosis (41). The physiological relevance of the plasticity and instability of helper T cell-like Treg cells (Th1-, Th2-, and Th17-like Tregs) and their role in the development of autoimmune diseases has yet to be clarified. Moreover, the molecular mechanisms and the environmental signals that trigger the development of helper T cell-like Treg cells in general and Th1-like Treg cells in particular are largely unknown (4). Our data suggest that endogenous GC act as an environmental signal to prevent Treg cell differentiation into Th1-like Treg cells and maintain Treg cell function in a T cell transfer model of colitis. The failure of GR-deficient Treg cells to respond to GC that are produced at increased levels during immune system activation (34), notably not only by the adrenals but also locally by the intestine itself (42), most likely leads to dysfunctional Treg cells in this disease model. Indeed, in another model of experimental colitis, dextran sodium sulfate (DSS)-induced colitis, endogenous circulating corticosterone levels were increased (43).

The proportion of functionally impaired GR-deficient Treg cells producing IFN-gamma more than doubled compared to control Treg cells but retained Foxp3, GITR and CD25 expression, whereas CTLA-4 was higher. IFN-gamma was shown by others to be involved in a functional defect of IFN-gamma^+^Foxp3^+^ Treg cells lacking Foxo1 (44). Foxo1-deficient Treg cells, which display a Th1-like phenotype, did not prevent disease in the T cell transfer colitis model. However, these Treg cells were partially protective when *Ifng* was deleted in addition to *Foxo1*. Phenotypically, Foxo1-deficient Treg cells expressed similar Foxp3, increased CD25 and marginally reduced CTLA-4 levels, compared to WT control Treg cells (44). In contrast, antigen (flagellin)-specific IFN-gamma^+^Foxp3^+^ Treg cells tested for suppression capacity in the same model of chronic colitis were found to maintain their regulatory function without reporting on Treg cell marker expression (45). Hence, it remains to be clarified on the one hand whether IFN-gamma^+^Foxp3^+^ Treg cells in general play a pathogenic or protective role in this setting and on the other hand which environmental signals and signaling pathways are responsible for driving the induction of IFN-gamma^+^Foxp3^+^ Treg cells. With respect to GR-deficient Treg cells, the generation of an animal model where IFN-gamma would be deleted together with the GR in Foxp3^+^ Treg cells (double-deficient Treg cells) would clarify whether Treg cell-derived IFN-gamma is causal for the dysfunction of GR-deficient Treg cells in experimental colitis.

The molecular mechanisms of how GR signaling prevents Treg cell plasticity and functional instability in transfer colitis are presently unknown. GC have been shown to upregulate *Foxp3* mRNA in CD4^+^ T cells of asthmatic patients (46) and in murine splenic CD4^+^CD25^high^ cells (11). Furthermore, the GR has been shown to interact with Foxp3 at the protein level as part of large multiprotein complexes (47). Conversely, Foxp3 binds the *Nr3c1* locus (47) and increases *Nr3c1* mRNA expression in thymic Treg cells (48), suggesting that both Foxp3 and GR are able to mutually regulate each other's expression levels and likely also their downstream targets. Supporting the view that the GR enhances Treg cell function is the observation that GC treatment of patients suffering from myasthenia gravis or multiple sclerosis not only improved clinical disease symptoms but also enhanced Treg cell function (12, 49) and inhibitory cytokine production (12), as compared to untreated patients.

Despite being defective under inflammatory conditions *in vivo*, the inhibitory potency of GR-deficient Treg cells in the *in vitro* suppression assays was not affected, irrespective of whether naïve Tcon or Tmem cells were used as target cells. The apparently contrasting results between *in vitro* and *in vivo* Treg cell functional assays have been previously reported in several other mouse models carrying a Treg cell specific deletion or mutation of a given gene (3). To explain this discrepancy, the current view is that Treg cells do not use one particular mechanism by which they exert their suppressor function, but rather use several pathways simultaneously, especially *in vivo* (3).

Collectively, our findings demonstrate that the GR is critical for Treg cell function under inflammatory conditions. Endogenous GC levels are typically increased in the course of immune and inflammatory responses and may, by GR signaling, counterregulate the acquisition of Th1 cell-like characteristics by Treg cells, such as the production of IFN-gamma, that would reduce their potency to suppress inflammation. Future studies will determine whether the loss of GR in Treg cells also accounts for increased Treg cell plasticity in other inflammatory and autoimmune disease models. If that would be the case, it may be justified to develop GC therapies for autoimmune and inflammatory disorders that specifically target Treg cells in order to reduce the strong side-effects of these hormones.

## Materials and Methods

### Mice

GRfl/fl mice (16) were bred on a C57BL/6 background to mice expressing Foxp3-YFP/Cre as a knocked-in YFP/iCre-recombinase fusion protein from the Foxp3 locus (17) to generate mice with GR-deficient Treg cells (Foxp3-YFP-Cre GRfl/fl mice). Foxp3-YFP-Cre mice were used as littermate controls for Foxp3-YFP-Cre GRfl/fl mice. Animals were housed in the Central Laboratory Animal Facilities of the Medical University of Innsbruck under standard light cycles and temperatures, and food and tap water were available *ad libitum*. C57BL/6 Foxp3-GFP reporter mice (50) were purchased from Jackson Labs (Bar Harbor, ME, USA) and served as CD4^+^ Tcon cell donors for the T cell transfer colitis experiments. RAG1^−/−^ mice were a kind gift from A. Moschen, Department of Internal Medicine II, Medical University Innsbruck. All animal experiments were performed in accordance with the Austrian “Tierversuchsgesetz” (BGBl. Nr. 501/1988 i.d.F. 162/2005) and have been granted by the Bundesministerium für Bildung, Wissenschaft und Kultur (bm:bwk).

### Flow Cytometry

Cell suspensions were prepared in KDS-BSS buffer containing 10% FCS. Cells were stained with combinations of the following antibodies for 20 min at 4°C: anti-CD4-PerCP/Cy5.5 (clone RM4-5) and anti-PD-1-PE (anti-CD279, clone J43) (both from eBiosciences, CA, USA); anti-CD8-PECy7 or anti-CD8-AF647 (clone 53-6.7), anti-B220-APC/Cy7 (clone RA3-6B2), anti-CD25-PE (clone 3C7) or CD25-BV421 (clone PC61), GITR-PE/Cy7 (clone YGITR765), CD45Rb-AF647 (clone C363-16A), LAP-PE (clone TW7-16B4), and CD223(LAG-3)-BV421 (clone C9B7W) (all from Biolegend, CA, USA); CD62L-PE (clone MEL-14) and CD44-BV510 (clone IM-7) (both from BD Biosciences; San Jose, CA). DAPI and Annexin-V (eBiosciences, CA, USA) were used to quantify or gate out apoptotic or dead cells.

For Foxp3 intracellular staining Foxp3/Transcription Factor Buffer set and anti-Foxp3-eF660 (clone FJK-16s) (both from eBiosciences) were used according to the manufacturer's instructions. For GR intracellular staining we used BD Cytofix and BD Cytoperm reagents (BD Pharmingen, CA, USA) and stained with anti-GR (clone D6H2L) (Cell Signaling, MA, USA), followed by a secondary antibody (goat anti-rabbit IgG AF647 (Invitrogen, OR, USA)). The same buffer set was used for cytokine staining using anti-IFN-gamma-PE (clone XMG1.2), IL-17-AF647 (clone TC11-18H10.1), anti-CTLA-4-PE (clone UC10-4B9; all from BioLegend), anti-IL-10-PE (clone JESS-16E3) and anti-Helios-APC (clone 22F6; both from eBiosciences).

### Cell Sorting

To obtain naïve conventional T cells (Tcon), CD4^+^Foxp3^−^(GFP^−^)CD25^−^CD45RB^high^ cells were sorted from spleen and/or mesenteric lymph nodes from Foxp3-GFP reporter mice. Memory T (Tmem) cells (CD4^+^Foxp3^−^(YFP^−^)CD44^+^CD62L^−^CD45RB^high^) were sorted from splenocytes from Foxp3-YFP-Cre mice. CD4^+^Foxp3^+^(YFP^+^)CD25^+^CD45RB^low^ Treg cells were isolated from Foxp3-YFP-Cre and Foxp3-YFP-Cre GRfl/fl mice. Cell sorting was performed using a FACSAria III cell sorter (Becton Dickinson) and purity of isolated cell populations was routinely at least 98%.

### Cell Culture

For the GC sensitivity test, single cell suspension of splenocytes (1 × 10^6^cells/mL) was incubated in flat bottom 96-well plates with corticosterone (Sigma, MO, USA) at 125 or 625 nM for 48 h and then analyzed for cell death as decribed (51). For TCR activation experiments, T cells (enriched by MACS) were seeded in anti-CD3 coated (5 μg/mL) 96-well round bottom plates and treated with soluble anti-CD28 (1 μg/mL; both antibodies from Biolegend) in the presence of 100 U/ml IL-2 (PreproTech, USA) in RPMI medium (supplemented with 50 uM beta-Mercaptoethanol, 100 U/mL Penicillin/Streptomycin, 2 mM L-Glutamine, 1 mM Na-Pyruvate, and non-essential amino acids), cultured for 48 h and then analyzed for activation status and cytokine production. For the *in vitro* T cell suppression assay, single cell suspensions were prepared from spleens and mesenteric lymph nodes from Foxp3-YFP-Cre and Foxp3-YFP-Cre GRfl/fl mice. 2 × 10^5^/mL Tcon cells or Tmem were stained with a cell proliferation dye (CPD-eF450) (eBioscience) and cultured together with irradiated (30 Gy) splenocytes (8 × 10^5^ cells/mL) in RPMI complete medium in 96-well round bottom plates. To induce cell proliferation, anti-CD3 (Biolegend, CA, USA) 0.5 μg/mL was added to the medium. In the indicated cases, different Treg cell concentrations were added to obtain Treg:Tcon ratios of 1:1, 1:2, 1:5, and 1:10. Cells were incubated for 72 h at 37°C and 5% CO_2_ and then analyzed for cell proliferation by Flow cytometry. For cytokine staining experiments, splenocytes were stimulated with 50 ng/mL PMA (Fluka Biochemika) and 1 mg/mL Ionomycin (Sigma) for 4 h. During the last 3 h of cell culture Monensin (Biolegend) was added.

### RNA Isolation and Quantitative RT-PCR (qPCR)

Total RNA was isolated from 1 × 10^5^ sorted cells using Quick-RNA MicroPrep kit (Zymo Research, CA, USA) and cDNA was synthesized using iScript cDNA Synthesis Kit (BioRad, CA, USA), according to the manufacturer's instructions. Real time PCR was performed using the following TaqMan Gene Expression Assays: GR (*Nr3c1*; Mm00433833_mH) and Actin-beta (*Actb;* Mm00607939_s1) and Luminaris Color Probe Master Mix (all from Thermo Fischer Scientific, MA, USA). Quantitative RT-PCR was analyzed using the StepOnePlus system (Applied Biosystems, Thermo Fischer Scientific, MA, USA) according to the manufacturer's instructions. The results were normalized to *Actb* expression and evaluated using the ΔΔCt relative quantification method.

### Antinuclear Antibodies (ANA) Detection

For detection of ANA we used Kallestad HEp-2 cell line 12-well slides from Bio-Rad (Hercules, California). Serum samples were diluted 1:50 and 1:100 and incubated on the slides according to the manufacturer's instructions. Fluorescence-labeled antibody AF488 donkey anti-mouse (Jackson ImmunoResearch Inc., West Baltimore Pike, West Grove, PA, USA) was used as a secondary antibody and slides were analyzed using a fluorescence microscope. Serum samples from MRL/lpr mice were used as positive controls for ANA detection.

### T Cell Transfer Model of Colitis

CD4^+^Foxp3^−^CD25^−^CD45RB^high^ Tcon cells were sorted from congenic C57BL/6 Foxp3-GFP mice and injected i.p. into 6 to 15-weeks-old C57BL/6 RAG1^−/−^ immunodeficient recipients (3 × 10^5^ cells/mouse). 1.5 × 10^5^ Foxp3-YFP-Cre or Foxp3-YFP-Cre GRfl/fl Treg cells (CD4^+^Foxp3^+^CD25^+^ CD45RB^low^) were co-injected i.p. where indicated. Mice were monitored every second day for wasting disease. Mice were sacrificed either when having lost >15% of their initial body weight or 4 weeks after cell transfer.

### Histology of Intestinal Inflammation

Samples of mid-colon were fixed in buffered 4% formalin solution. Three millimeter paraffin-embedded sections were cut and stained with hematoxylin and eosin. Tissues were evaluated semi-quantitatively and assigned a grade of 0 to 4 in a blinded fashion. Grade 0: no changes observed, grade 1: discrete increased inflammatory cells in the lamina propria with granulocytes in the lamina epithelialis, grade 2: as grade 1 with scattered erosions of the mucosa, grade 3: increased inflammatory cells in the lamina propria and scattered crypt abscesses, grade 4: all signs of grade 3 plus more than 3 crypt abscesses per colon circumference in the scanning magnification.

### Western Blot

Cellular subsets (3.5 × 10^5^ cells/subset) were resuspended in Laemmli sample buffer and heated in boiling water for 5 min. Total proteins were loaded on 10% Bis-Tris acryl-amide gels and blotted on AmershamTM HybondTM-ECL nitrocellulose membranes (GE Healthcare, Little Chalfont, UK). Rabbit anti-mouse GR (clone D6H2L) (Cell Signaling, MA, USA) and rabbit anti-mouse AKT (Cell signaling Technology, Danvers, MA) were used for protein detection. All primary antibodies were diluted in 5% BSA in PBST and blots were incubated overnight at 4°C.

### ELISA for Serum Corticosterone

Serum samples from Foxp3-YFP-Cre and Foxp3-YFP-Cre GRfl/fl mice were collected between 9 and 10 a.m. and analyzed for corticosterone content by ELISA (Enzo Life Sciences, CH) according to the manufacturer's instructions.

### DNA Methylation Analysis

Genomic DNA was isolated from sorted CD4^+^CD25^−^CD45RB^high^ conventional T cells as well as CD4^+^Foxp3^+^CD25^+^ Treg cells sorted from WT Foxp3-GFP reporter, Foxp3-YFP-Cre mice and Foxp3-YFP-Cre GRfl/fl mice using the DNeasy blood and tissue kit (Qiagen, Hilden, Germany). Bisulfite modification was performed using the EZ DNA Methylation-Gold Kit (ZymoResearch). MethyLight PCR analysis and the calculation of the percentage of methylated reference (PMR) were done as described previously (52, 53). Two Foxp3 assays (one reaction for DNA methylation analysis and one for internal reference, with a mean distance of −2.226 base pairs, or −3.866 base pairs respectively, to the transcription start site) were determined with the assistance of the computer program Primer Express version 2.0.0 (Applied Biosystems, Foster City, CA, USA). Primers used have been described previously (53).

### Statistics

Estimation of statistical differences between groups was carried out using the unpaired Student's *t*-test or two-way ANOVA test, where appropriate. A chi-square test was used to test for differences between groups regarding prevalence of ANA. *P* ≤ 0.05 were considered to indicate statistically significant differences. ^ns^*p* ≥ 0.05; ^*^p < 0.05; ^**^p ≤ 0.01; ^***^p < 0.001; and ^****^p < 0.0001.

## Data Availability

All datasets generated for this study are included in the manuscript and/or the supplementary files.

## Author Contributions

LR-R designed and performed most experiments, statistical analysis, prepared figures, and wrote paper. ST designed and performed experiments. LvR, MT, and HF performed experiments. MD performed histopathological assessment. HR interpreted data and wrote sections of the paper. AV designed research, interpreted data and wrote sections of the paper. DT and GW designed research, interpreted data, wrote paper, and conceived study.

### Conflict of Interest Statement

The authors declare that the research was conducted in the absence of any commercial or financial relationships that could be construed as a potential conflict of interest.
