# Supplementary material for: Glucocorticoid Receptor-Deficient Foxp3+ Regulatory T Cells Fail to Control Experimental Inflammatory Bowel Disease
Source: Front Immunol. 2019 Mar 18;10:472. doi: 10.3389/fimmu.2019.00472 (PMC6431616; doi:10.3389/fimmu.2019.00472)
Supplement: Supplementary file 1 [file Presentation_1.pptx]

## Slide 1
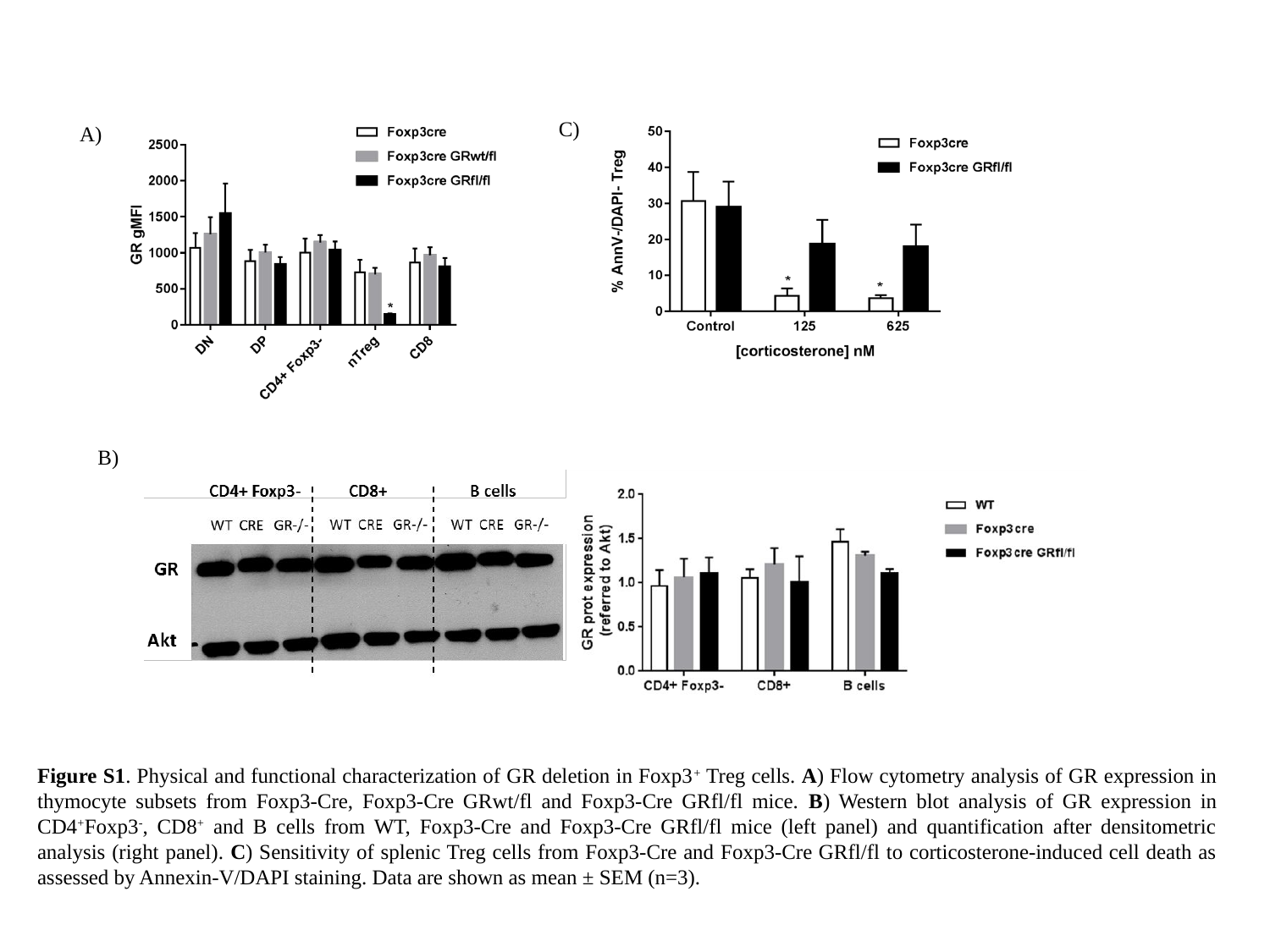

C)
A)
B)
Figure S1. Physical and functional characterization of GR deletion in Foxp3+ Treg cells. A) Flow cytometry analysis of GR expression in thymocyte subsets from Foxp3-Cre, Foxp3-Cre GRwt/fl and Foxp3-Cre GRfl/fl mice. B) Western blot analysis of GR expression in CD4+Foxp3-, CD8+ and B cells from WT, Foxp3-Cre and Foxp3-Cre GRfl/fl mice (left panel) and quantification after densitometric analysis (right panel). C) Sensitivity of splenic Treg cells from Foxp3-Cre and Foxp3-Cre GRfl/fl to corticosterone-induced cell death as assessed by Annexin-V/DAPI staining. Data are shown as mean ± SEM (n=3).

## Slide 2
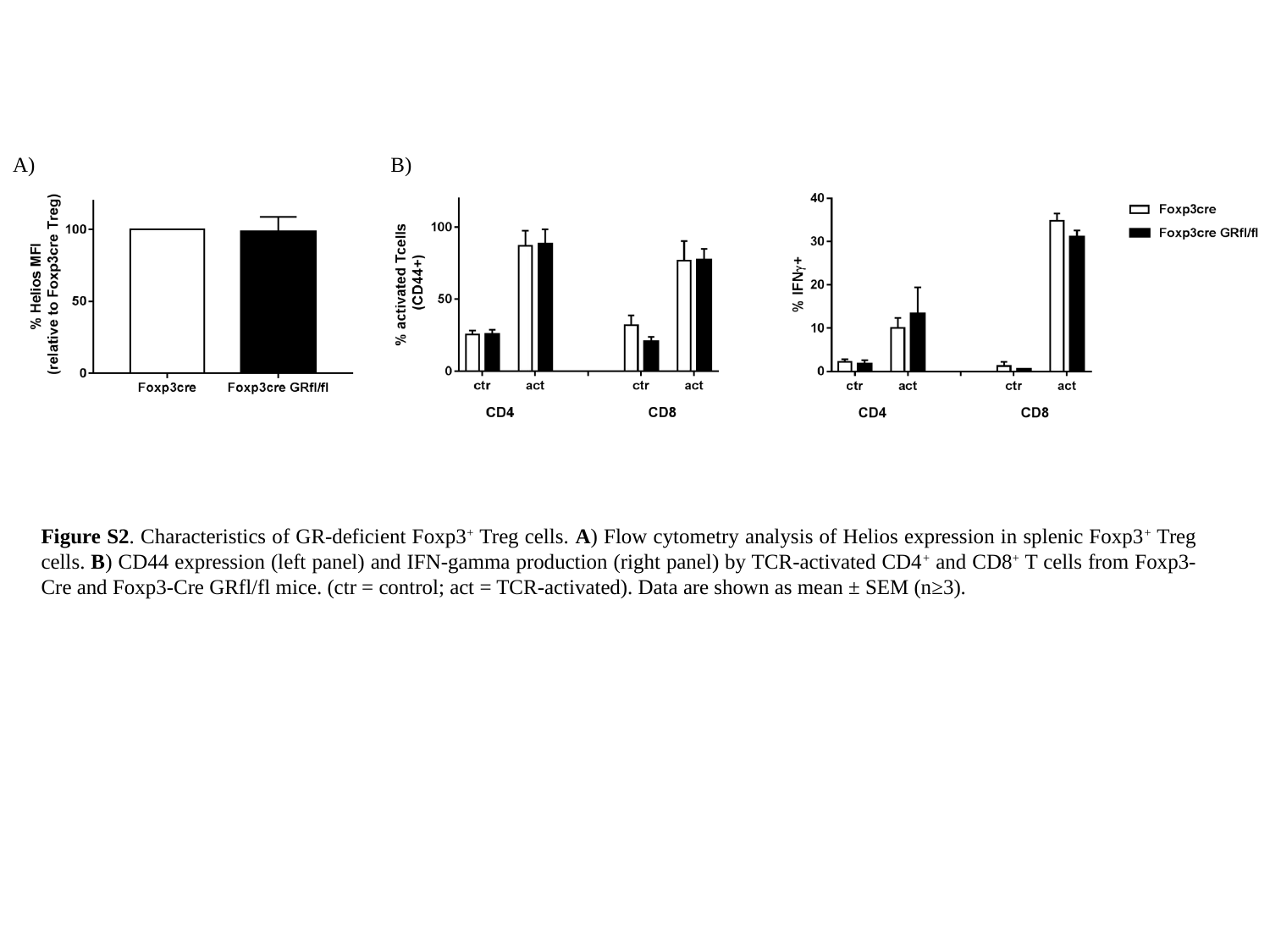

B)
A)
Figure S2. Characteristics of GR-deficient Foxp3+ Treg cells. A) Flow cytometry analysis of Helios expression in splenic Foxp3+ Treg cells. B) CD44 expression (left panel) and IFN-gamma production (right panel) by TCR-activated CD4+ and CD8+ T cells from Foxp3-Cre and Foxp3-Cre GRfl/fl mice. (ctr = control; act = TCR-activated). Data are shown as mean ± SEM (n≥3).

## Slide 3
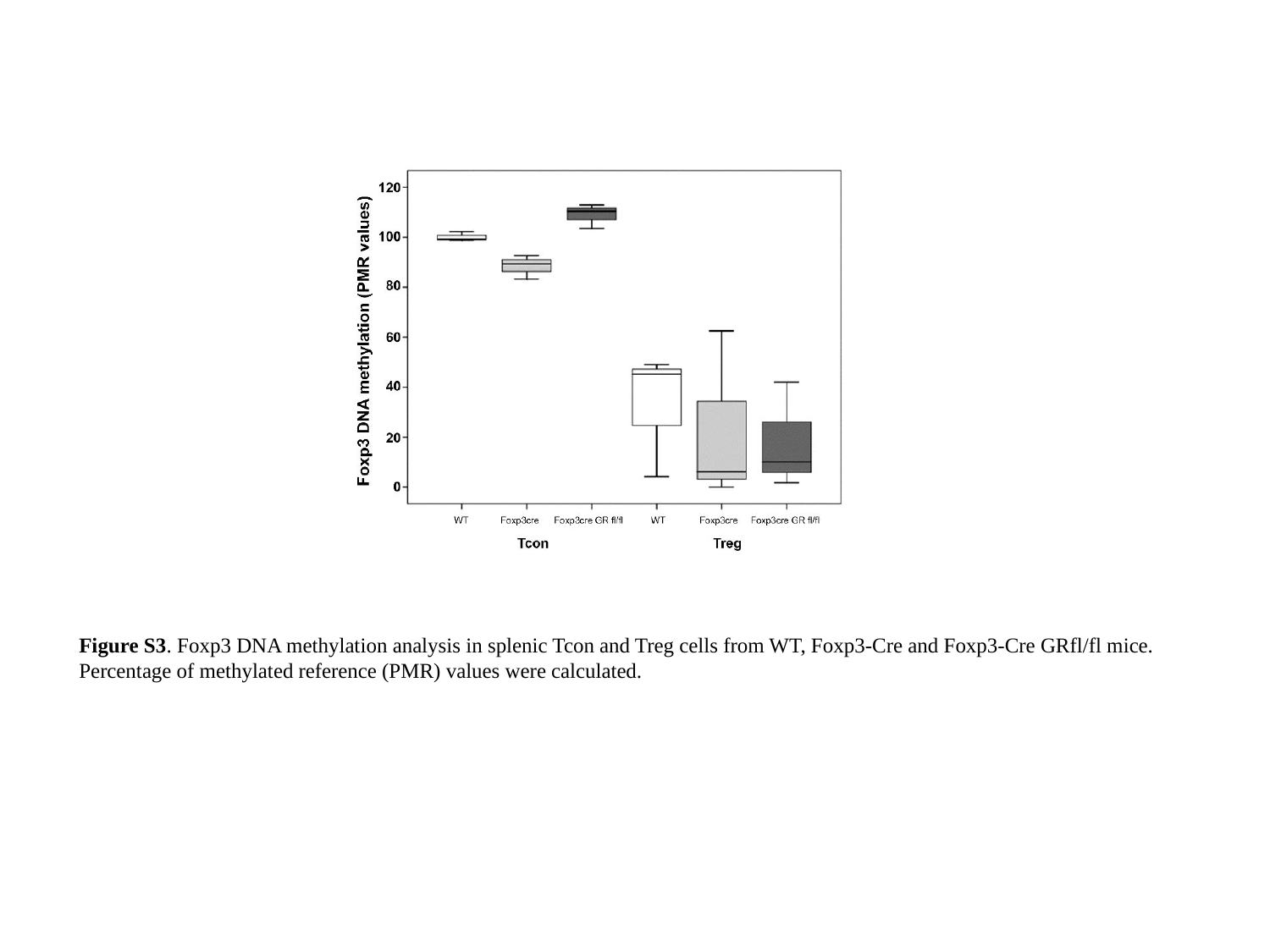

Figure S3. Foxp3 DNA methylation analysis in splenic Tcon and Treg cells from WT, Foxp3-Cre and Foxp3-Cre GRfl/fl mice. Percentage of methylated reference (PMR) values were calculated.

## Slide 4
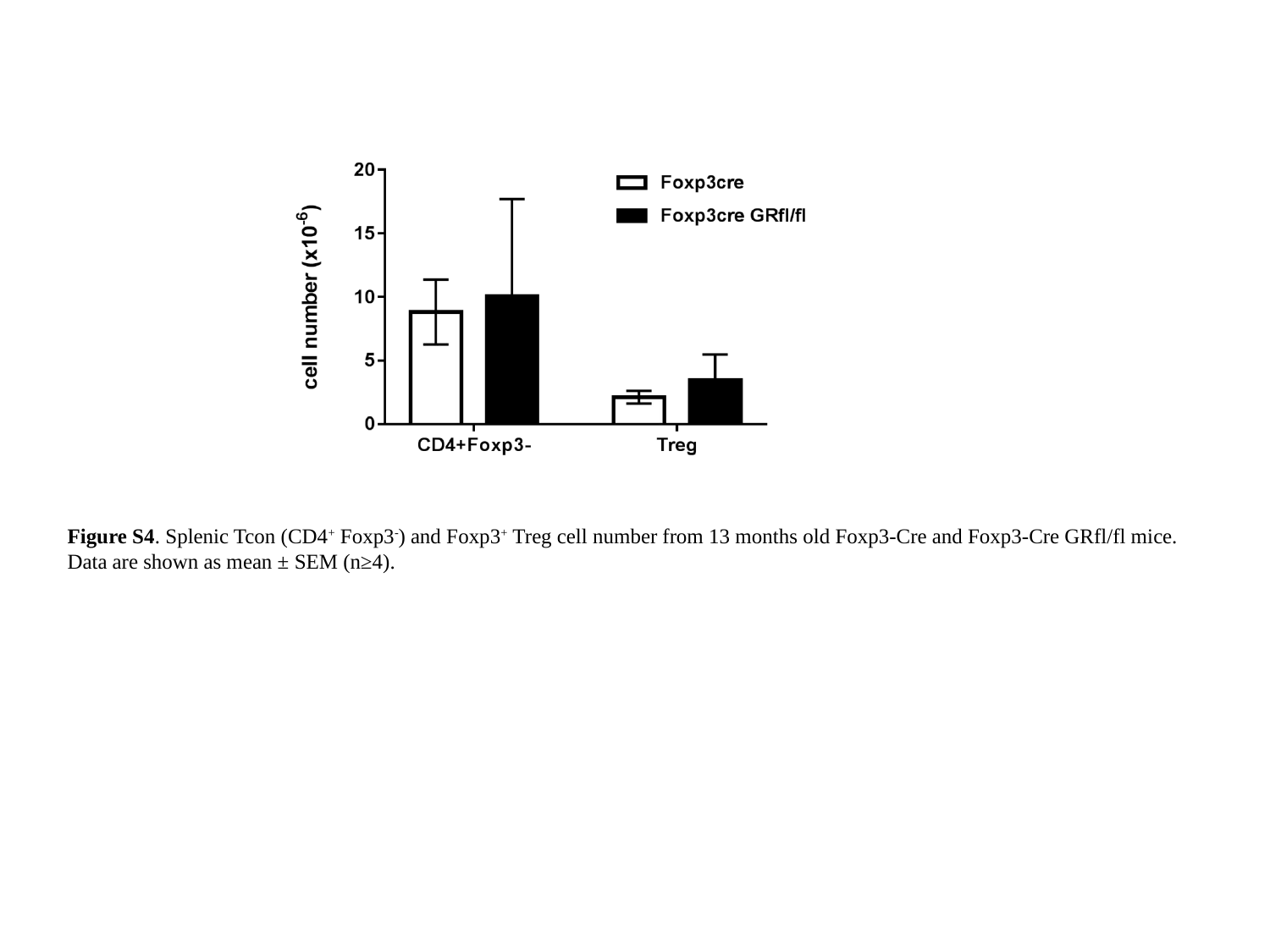

Figure S4. Splenic Tcon (CD4+ Foxp3-) and Foxp3+ Treg cell number from 13 months old Foxp3-Cre and Foxp3-Cre GRfl/fl mice. Data are shown as mean ± SEM (n≥4).

## Slide 5
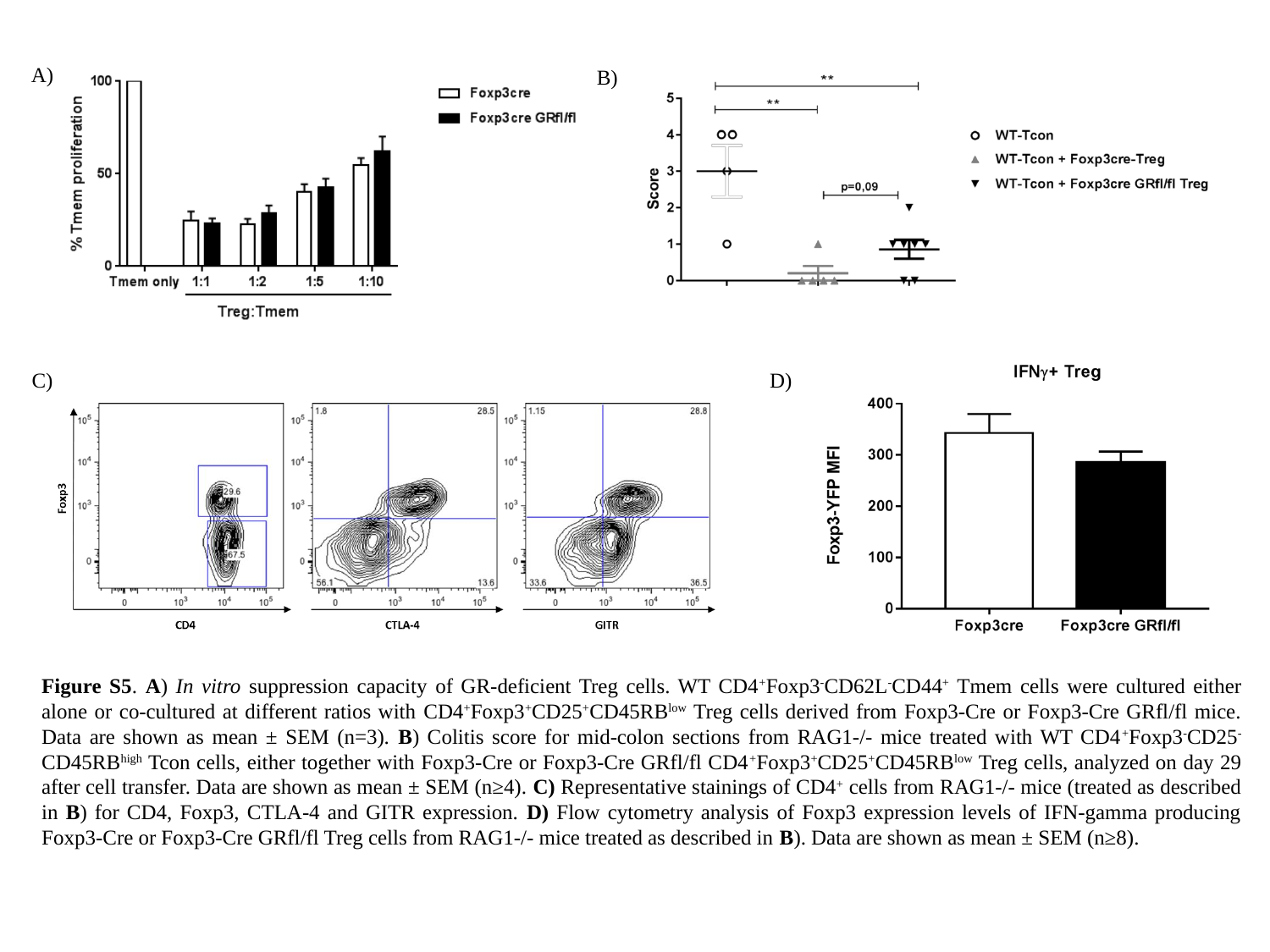

A)
B)
D)
C)
Figure S5. A) In vitro suppression capacity of GR-deficient Treg cells. WT CD4+Foxp3-CD62L-CD44+ Tmem cells were cultured either alone or co-cultured at different ratios with CD4+Foxp3+CD25+CD45RBlow Treg cells derived from Foxp3-Cre or Foxp3-Cre GRfl/fl mice. Data are shown as mean ± SEM (n=3). B) Colitis score for mid-colon sections from RAG1-/- mice treated with WT CD4+Foxp3-CD25-CD45RBhigh Tcon cells, either together with Foxp3-Cre or Foxp3-Cre GRfl/fl CD4+Foxp3+CD25+CD45RBlow Treg cells, analyzed on day 29 after cell transfer. Data are shown as mean ± SEM (n≥4). C) Representative stainings of CD4+ cells from RAG1-/- mice (treated as described in B) for CD4, Foxp3, CTLA-4 and GITR expression. D) Flow cytometry analysis of Foxp3 expression levels of IFN-gamma producing Foxp3-Cre or Foxp3-Cre GRfl/fl Treg cells from RAG1-/- mice treated as described in B). Data are shown as mean ± SEM (n≥8).

## Slide 6
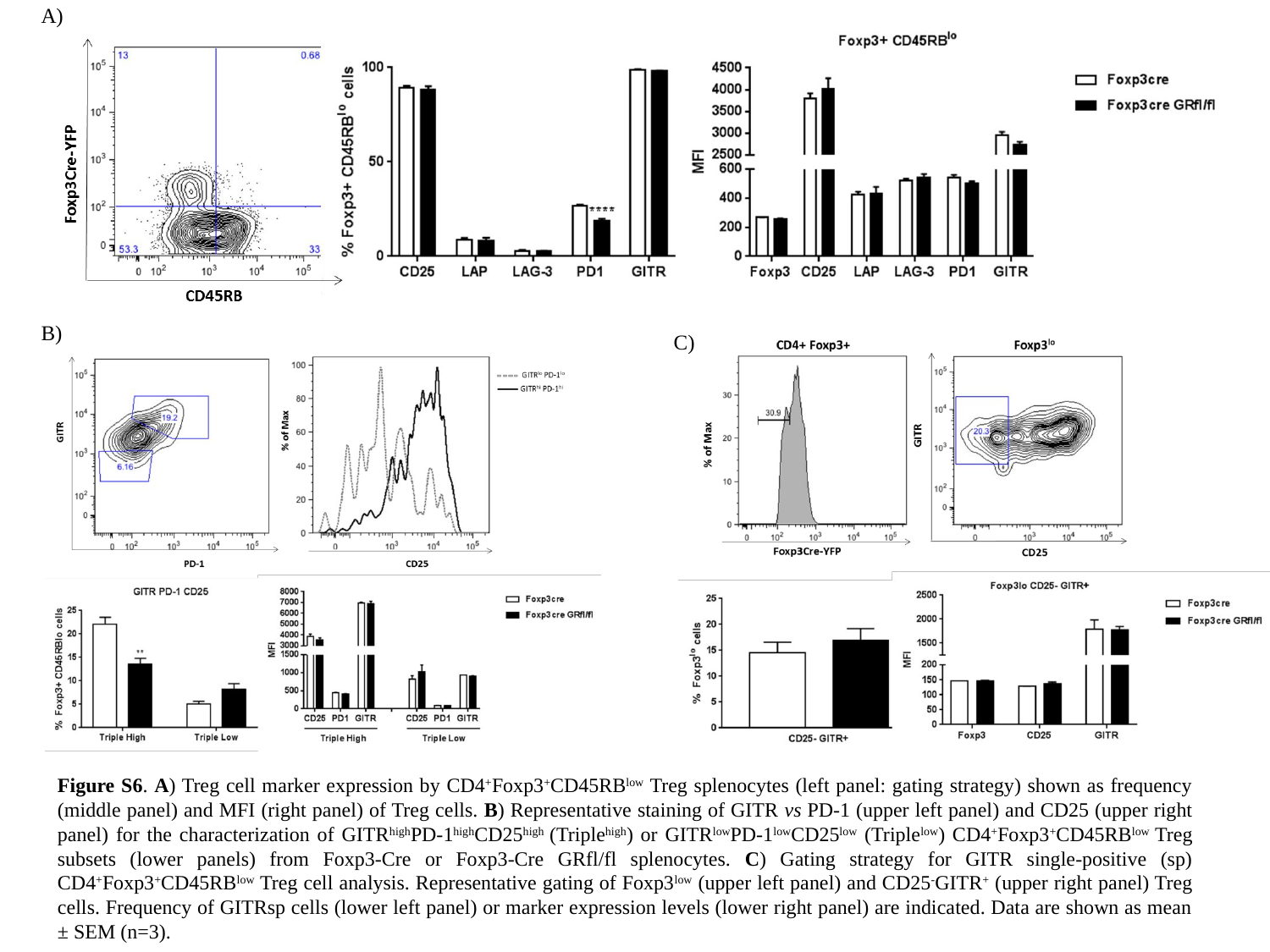

A)
B)
C)
Figure S6. A) Treg cell marker expression by CD4+Foxp3+CD45RBlow Treg splenocytes (left panel: gating strategy) shown as frequency (middle panel) and MFI (right panel) of Treg cells. B) Representative staining of GITR vs PD-1 (upper left panel) and CD25 (upper right panel) for the characterization of GITRhighPD-1highCD25high (Triplehigh) or GITRlowPD-1lowCD25low (Triplelow) CD4+Foxp3+CD45RBlow Treg subsets (lower panels) from Foxp3-Cre or Foxp3-Cre GRfl/fl splenocytes. C) Gating strategy for GITR single-positive (sp) CD4+Foxp3+CD45RBlow Treg cell analysis. Representative gating of Foxp3low (upper left panel) and CD25-GITR+ (upper right panel) Treg cells. Frequency of GITRsp cells (lower left panel) or marker expression levels (lower right panel) are indicated. Data are shown as mean ± SEM (n=3).
